# Whole exome sequencing reveals a homozygous nonsense mutation in *HEXA* gene leading to Tay-Sachs disease in Saudi Family

**DOI:** 10.12669/pjms.36.6.2579

**Published:** 2020

**Authors:** Muhammad Imran Naseer, Angham Abdulrahman Abdulkareem, Mohammed Mohammed Jan, Adeel G. Chaudhary, Mohammad H. Al-Qahtani

**Affiliations:** 1 Muhammad Imran Naseer, Center of Excellence in Genomic Medicine Research, Department of Medical Laboratory Technology, Faculty of Applied Medical Sciences, King Abdulaziz University, 21589, Jeddah, Saudi Arabia; 2 Angham Abdulrahman Abdulkareem, Biochemistry Department, Faculty of Science, King Abdulaziz University, 21589, Jeddah, Saudi Arabia; 3 Mohammed Mohammed Jan, Department of Pediatrics, Faculty of Medicine, King Abdulaziz University, 21589, Jeddah, Saudi Arabia; 4 Adeel G. Chaudhary, Center of Excellence in Genomic Medicine Research, Department of Medical Laboratory Technology, Faculty of Applied Medical Sciences, Center for Innovation in Personalized Medicine, King Abdulaziz University Hospital, Jeddah Saudi Arabia. King Abdulaziz University, 21589, Jeddah, Saudi Arabia; 5 Mohammad H. Al-Qahtani, Center of Excellence in Genomic Medicine Research, Department of Medical Laboratory Technology, Faculty of Applied Medical Sciences, King Abdulaziz University, 21589, Jeddah, Saudi Arabia

**Keywords:** *HEXA* gene, Tay-Sachs disease, Neurodegenerative disorder inherited, Saudi Family

## Abstract

**Objective:**

To study the causative variants in affected member of a Saudi family with Tay-Sachs disorder. This disorder includes paralysis, decreasing in attentiveness, seizures, blindness, motor deterioration progresses rapidly leading to a completely unresponsive state and a cherry-red spot visible on the eye.

**Methods:**

Whole exome sequencing (WES) and Sanger sequencing was performed to study the variant leading to the disease.

**Results:**

WES data analysis and Sanger sequencing validation, identifies a homozygous nonsense mutation c.1177C>T, p.Arg393Ter as a result in protein change. This mutation was also studied in 100 unrelated healthy controls.

**Conclusions:**

We detected homozygous mutation in *HEXA* gene that may lead to cause Tay-Sachs disorder. Moreover, explain the possibility that *HEXA* gene may play important role for multiple aspects of normal human neurodevelopment.

## INTRODUCTION

Tay-Sachs disease (TSD) is a deadly genetic disorder; affect the nerve cells in the brain and spinal cord. This disease is inherited in an autosomal recessive pattern. Infants are affected most severely, by the age of 3-5 months. This disease characterized by symptoms such as paralysis, decreasing in attentiveness, seizures, blindness, decreased muscle strength and inability to move. This disease characterized by the increase of GM2 ganglioside in neurons at the beginning of the fatal life leading to toxicity.

This accumulation caused by the absence of A (acidic) isozyme of lysosomal β-hexosaminidase who has the ability to reduce fatty acid derivatives called GM2 ganglioside in concert with the small glycolipid transport protein called GM2A activator, which act as a cofactor. The ganglioside is stored in the form of concentrically arranged lamellae known as membranous cytoplasmic bodies (MCBs). The accumulation of ganglioside at the terminal phase in the patient with TSD cause a ballooned filled with MCBs in their neurons and changing in the cellular architecture.^1^ There’s no curative treatment for TSD. Diagnosis is poor and the neural dysfunction progresses without response to medication.^2^

*HEXA* gene localized on chromosome 15q23-q24, which encodes α-subunit of the lysosomal enzyme β -N-acetylhexosaminidase A. The enzyme is a dimer composed of one α- and one β-subunit.^3^,^4^ The *HEXA* gene has been isolated and characterized. It is 35 kb long and contains 1,587 bp of coding sequence separated into 14 exons.^4^ A promoter region has been predicted from sequence analysis but has not been confirmed experimentally. The elucidation of the gene structure has made possible the identification of a number of mutations in the *HEXA* gene.^4^

In this study exome sequencing was done for the affected members in a Saudi family to study the changes. We found a homozygous nonsense variant in *HEXA* gene (OMIM:606869;NM_000520.4:C.1177C>T, p.Arg393Ter), this mutation case TSD Syndrome. WES data analysis, was validated by Sanger sequencing analysis that identified the same variant in the heterozygous state in both parents and unaffected sibling which is known make them carriers.

## METHODS

**Fig.1:**
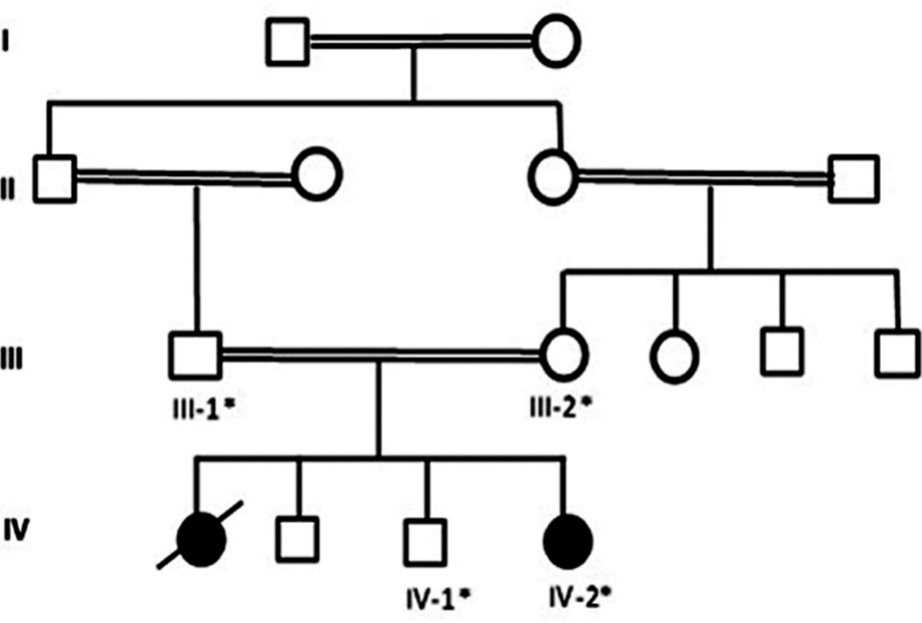
Pedigree of the family was drawn after having details from the parents. The * sign represent the available samples for the study.

Blood samples from affected people were collected according to an appropriate local ethical rules and specific strategies from the king Abdul-Aziz University Hospital, Jeddah. Written approval was obtained from all members according to the Helsinki Declaration. This study was accepted from the Center of Excellence in Genomic Medicine Research, King Abdulaziz University. In order to eliminate any non-genetic cause and to estimate the severity and laterality of the disease Magnetic resonance imaging and Electroencephalogram were made. A peripheral blood sample was taken from the patient to extract the DNA. A complete history detailed was taken from the family, after that the pedigree was drawn as shown in Fig.1. Further the samples for WES were prepared according to instruction using illumina NextSeq instrument with 2x76 paired end reads.

The blood samples had been collected from all four members of the family and a control one hundred unrelated healthy Saudi people. The affected individual went through a different medical examination at king Abdul-Aziz University Hospital

### Clinical history

The proband (IV-2) is a Saudi girl with age of two years, consanguineous, fourth child of three siblings. Her clinical manifestations include loss of vision, inability to walk, seizures and suggestive an undiagnosed neurological disorder.

### Methods

### Whole exome sequencing (WES)

DNA from affected individual is enriched for the whole coding region and splice site junctions of the genes on the used panel. The products are sequenced on an illumine NextSeq instrument with 2x76 paired end reads. The sequence is compared to a reference sequences based on human genome build GRCH37/UCSC hg19. Capillary sequencing is used to check out the relevant variants with clinical or uncertain significance. All sequence alterations are defined by using the Human Genome Variation society nomenclature guidelines. By using the gene-specific filtering data were analyzed. Various bioinformatics analyses were carried to identify causative variant in *HEXA* gene. Variants obtained from sequencing were filtered to include only novel/rare (MAF=0.01%), functional (predicted damaging by SIFT/Polyphen), present in the heterozygous/homozygous state in the affected individual literature search and database queries were performed to limit the variants to genes relevant to the clinical history reported.

### Sanger sequencing

To validate the WES data Sanger sequencing was done as explained previously.^5^ Sequence trace file was aligned to the corresponding reference sequence using the Software as explained previously.^5^ Furthermore this mutation was ruled out in 100 control samples.

## RESULTS

**Fig.2:**
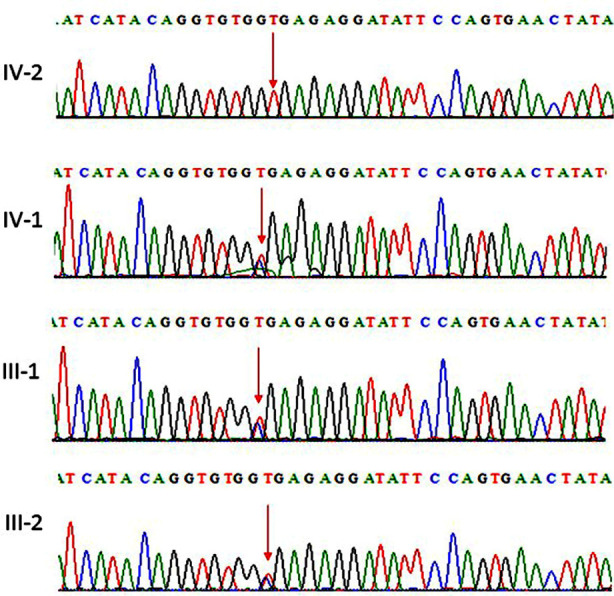
Sanger sequence analysis: (III-1 and III-2) are carrier parents and carrier brother (IV-1) showing T and A in heterozygous state, while (IV-2) are affected child showing only homozygous T in *HEXA* gene.

WES was done for the sample obtained from patient with 2years old. The results revealed a homozygous nonsense variant in *HEXA* gene (OMIM:606869; NM_000520.4:C.1177C>T, p.Arg393Ter). Sanger sequencing identified the same variant in the heterozygous state in both parents and unaffected sibling who is known make them carriers as shown in Fig.2. The pattern of family segregation supports the pathogenicity of this variant which is a known mutation reported in ClinVar.

## DISCUSSION

In this study we detected homozygous nonsense variant in *HEXA* gene C.1177C>T, p.Arg393Ter this would expect to lead to TSD. The patient in this study share a common clinical image, which includes loss of vision, inability to walk, seizures and suggestive an undiagnosed neurological disorder. A study in (1975) by Beutler et al. decided that TSD is acquired by changing in the *HEXA* gene causing a dysfunctional in Hexosaminidase A (alpha polypeptide) enzyme.^6^

Total of 78 mutations in the *HEXA* gene was reported containing 65 point mutation, 13 indel mutations along with 45 missense mutations leading to the Tay-Sachs caused by point mutations.^7^ Also they found six nonsense mutation and 14 splice site lesions caused by point mutation. In addition to that frame shift mutation can led to TSD, eight cases were reported (6 deletion, 2 insertion), one of the insertion cases within exon 11 four base pair were inserted, this type of mutation showed in 80% in the carriers of TSD from the Ashkenazi Jewish population. A lagre deletion in exon1 found in French Canadian patient, maybe the main cause of TSD, while Most of the other changing are limited to single pedigrees.^7^ A study was published in (2002) by McGinniss et al. recognized 8 novel mutation in the *HEXA* gene, in addition to that, they found 31 mutation in 49 subjects.^8^ Tanaka et al. (1990) studied 7 patients with TSD caused by a specific enzymologic characteristic of B1 variant of TSD. This variation has been considered a normal catalytic activity on certain synthetic substrates, but defective catalytic activity against natural substrates, including GM2 gangliosides.^9^ More or less all cases, but one from Czechoslovakia, have exactly the same arg178-to-his mutation. The Czechoslovakian case had change in the same codon but the alteration of nucleotide 532 from C to T change the amino acid from Arginine to Cytosin and this cause a protein dysfunction. As well as 136 consanguineous families screened by next generation sequencing they found that 90% Iranian and less than 10% Turkish or Arabic have syndromic or nonsyndromic in the forms of autosomal recessive intellectual disability.^9^ A missense mutation was discovered in a family by Najmabadi et al. (2011) in which the first-cousin parents had 8 children 3 of them has moderate symptoms while the rest were healthy.^10^ Here in this study we report a homozygous nonsense mutation c.1177C>T, p.Arg393Ter as a result in protein change in *HEXA* gene for the first time in Saudi Family. Our findings further expand the role of WES in efficient tool for disease diagnosis in Arab families and explained that the mutation in *HEXA* gene may plays an important role and it will further help to understand the development of TSD syndrome.

### Authors’ Contributions:

**MIN and AGC** designed the experiments.

**AAA, MMJ and MIN** conducted the experiments.

**MHA, MIN, MMJ and AAA** analyzed the data.

**MIN, AAA and AGC** wrote the paper.

All authors contributed to the editing of the paper, the scientific discussions and are responsible for integrity of the study.
